# Multidrug-resistant *Campylobacter jejuni, Campylobacter coli and Campylobacter lari* isolated from asymptomatic school-going children in Kibera slum, Kenya

**DOI:** 10.12688/f1000research.21299.2

**Published:** 2020-09-04

**Authors:** Nduhiu Gitahi, Peter B. Gathura, Michael M. Gicheru, Beautice M. Wandia, Annika Nordin

**Affiliations:** 1 Department of Public Health, Pharmacology & Toxicology, University of Nairobi, Nairobi, 00100, Kenya; 2 Department of Zoological Sciences, Kenyatta University, Nairobi, 00100, Kenya; 3 Department of Energy and Technology, Swedish University of Agricultural Science, Uppsala, Sweden

**Keywords:** Multidrug, resistance, Campylobacter, genes, asymptomatic

## Abstract

**Background:** The objective of this study was to determine the prevalence of thermophilic *Campylobacter *spp. in asymptomatic school-going children and establish the antibiotic resistance patterns of the isolates towards the drugs used to treat campylobacteriosis, including macrolides, quinolones and tetracycline. *Campylobacter *spp. are a leading cause of enteric illness and have only recently shown resistance to antibiotics.

**Methods:** This study isolated *Campylobacter *spp., including *Campylobacter coli*, *Campylobacter jejuni* and *Campylobacter lari*, in stool samples from asymptomatic school-going children in one of the biggest urban slums in Kenya. The disc diffusion method using EUCAST breakpoints was used to identify antibiotic-resistant isolates, which were further tested for genes encoding for tetracycline resistance using primer-specific polymerase chain reaction.

**Results:** In total, 580 stool samples were collected from 11 primary schools considering both gender and age. Subjecting 294 biochemically characterized *Campylobacter *spp. isolates to genus-specific PCR, 106 (18.27% of stool samples) isolates were confirmed *Campylobacter *spp. Out of the 106 isolates, 28 (4.83%) were *Campylobacter* *coli*, 44 (7.58%) were *Campylobacter jejuni* while 11 (1.89%) were *Campylobacter* *lari*. *Campylobacter jejuni* had the highest number of isolates that were multi-drug resistant, with 26 out of the 28 tested isolates being resistant to ciprofloxacin (5 mg), nalidixic acid (30 mg), tetracycline (30 mg) and erythromycin (15 mg).

**Conclusions:** In conclusion, asymptomatic school going children in the study area were found to be carriers of multidrug resistant *Campylobacter coli*, *Campylobacter jejuni* and *Campylobacter lari* at 84%. A one-health approach, which considers overlaps in environment, animals and human ecosystems, is recommended in addressing multidrug resistane in Campylobacter, since animals are the main reservoirs and environmental contamination is evident.

## Introduction

*Campylobacter* spp. infection is a leading cause of enteric illness ^1, 2^, manifesting as mild-to-severe diarrhoea with watery loose stool that is often followed by bloody diarrhoea ^3^. Infections also manifest as meningitis, pneumonia, miscarriage, severe form of Guillain-Barre syndrome (GBS) and reactive arthritis (ReA) and irritable bowel syndrome (IBS) ^3– 5^. Isolation of pathogenic *Campylobacter* spp. from asymptomatic children would be as a result of the pathogens not expressing the virulence factor cytolethal distending toxin, which is able to induce host cell apoptosis ^6^. Pathogenesis could also be influenced by host immune system and pathogens adaptation strategies ^6^. Other factors like motility and chemotaxis affect effective *Campylobacter* colonization and pathogenesis; these have been shown to vary in mutants ^7^.

*Campylobacter* spp. are found in the intestinal tract of wild and domestic animals, particularly in birds, asymptomatically as temporal carriers but causing illness in humans ^3^. The bacteria can survive up to five months at -20°C but die off in a few days at room temperature ^5, 8, 9^. Campylobacter spp is vulnerable to air exposure, drying, low pH and heating ^3^. Three species, namely *C. jejuni, C. coli* and *C. lari*, account for 99% of human *Campylobacter* spp. isolates, with *C. jejuni* accounting for 90% of the isolates. *C. fetus* and *C. upsaliensis* have also been isolated in humans ^10– 12^.

Distinguishing between *Campylobacter species* using phenotypic methods is difficult; however, genotypic methods have been developed that are capable of differenting the species. This has enabled more elaborate epidemiological understanding of Campylobacteriosis, identification of the sources and routes of infection ^13, 14^. The use of multiplex PCR methods has resulted in cheap, rapid and sensitive genetic identification of Campylobacter spp ^15^. 

It was not until the last two decades that *Campylobacter* spp. was shown to exhibit multidrug resistance (MDR). Before that, the bacteria were considered to be susceptible ^16^. Tetracycline is one of the antimicrobial agents against which *Campylobacter* spp. have shown resistance. In *Campylobacter* spp. *,* tetracycline resistance has been reported to be mediated by more than one tetracycline resistance ( *tet*) genes. The *tet*(O) and *tet*(S) genes are the ribosomal protection protein and plays the primary part in tetracycline resistance in *C. jejuni* and *C. coli* ^17, 18^. This is transferred as a plasmid encoded gene ^19^ or as non-self-mobile form. The *tet*(A) gene encodes the 46 kDa membrane-bound efflux protein. This protein carries tetracycline from the cell membrane and its first known resistance role in *Campylobacter* spp. was reported in 2014 ^16^. 

In *Campylobacter* spp. resistance to quinolones is mainly due to a single point mutation in the quinolone resistance determination region of *gyrA* gene (QRDR) ^20, 21^, at amino acid 86 by replacement of Thr by Ile ^22^. Occasionally, mutation in topoisomerase IV (ParC) results to resistance against quinolones. Other amino acids substitutions have been reported by Piddock *et al*. and others ^23– 26^. In *Campylobacter* there has been no documented mutational change to the *gyrB* subunit gene in relation to resistance against quinolones; however, Piddock *et al*. ^22^, and Changkwanyeun *et al*. ^27^ noted that resistance to ciprofloxacin in *Campylobacter* is mediated by mutations on the *gyrA* gene.

## Methods

### Study area and background

The study was carried out at primary schools located at Kibera informal settlement, Nairobi County, Kenya in July 2015. Kibera is located at an altitude of 1670 m above sea level, at latitude 36°50’ east and longitude 1°17’ south, about 140 km south of the equator. Kibera is located 5 km South of Nairobi Central Business District (CBD), the Capital of Kenya. Kibera is divided into 9 official villages. The average living place is 3 m ^2^, with an average of 5 persons per place. The study site presents a population with diverse enteric infections ^28^. In total, 11 primary schools with pupil population ranging from 120 to 189 were randomly sampled and, 40 to 80 stool samples collected from pupils in each school, depending on the school population, making a total of 580 stool samples. With a known prevalence of 40.7% of soil transmitted helminths in school going children in urban Kenya, the formula by Martin *et al*. (1998) was used to determine the desired minimum sample size. The schools were distributed in five administrative villages, namely Lindi, Silanga, Laini Saba, Gatwekera and Mashimoni. Participants’ parents provided written consent through the care givers. This was done during parents’ school meetings, where parents were informed of the intended study and its benefits, those who agreed their children to participate were issued with consent forms for them to sign and return to their class teacher. Only those who their parents consented participated in the study.

Research clearances were given by National Commission for Science, Technology and Innovation (research clearance permit No. 3756) and ethical clearance (PKU/278/1274) was granted by Kenyatta University Ethical Review Committees.

***Campylobacter spp. culture.*** In the laboratory, 5 g of freshly collected faecal sample was pre-enriched by suspending the faeces in 45 ml buffered peptone water (BPW) (Oxoid, Hampshire, England) and incubating the suspension at 42°C for 18 hours in a 50-ml closed culture tube. The pre-enrichment was inoculated onto modified campylobacter charcoal-cefoperazone deoxycholate (mCCDA) agar plates with supplement (polymyxin B 2500IU, rifampincin 5 mg, trimethoprim 5 mg and cycloheximide 50 mg) using a sterile swab and the plates incubated at 45°C for up to 48 hours under anaerobic conditions. The mCCDA culture media (Oxoid, Hampshire, England) was prepared according to manufacturer’s instructions and stored at 4°C until use. Micro-aerobic conditions were achieved by adding a 21.3-g sachet of CampyGen ^TM^ 3.5 L (Oxoid, Hampshire, England) in an anaerobic jar with the cultures resulting to a maximum of 13.2% O _2_ within 24 hours and 9.5% CO _2_ in 1 hours. After 24 hours of incubation, the plates were checked for characteristic growth and plates without growth were re-incubated for an additional 24 hours. Characteristic colonies (grey/white or creamy grey in colour with moist appearance) were examined and counted. Distinct colonies were harvested and tested for oxidase and peroxidase breakdown, by picking a portion of distinct colony with a sterile wire loop and placing it on a drop of 30% hydrogen peroxide on a clean microscope slide. Production of effervescent air bubbles was recorded as peroxidases positive. The same colonies were tested for cytochrome oxidase enzyme production by placing a portion of the test colony onto oxidase paper impregnated with NNN’N’ tetramethyl-p-phenylene-diamine dihydrochloride (Oxoid, Basingstoke, UK). Purple colour change was recorded as positive reaction. Reactive colonies were processed for DNA and a portion stored in skimmed milk at -80°C for further characterization.

***DNA preparation from bacteria colonies and multiplex PCR.*** For DNA extraction, three distinct colonies from pure bacteria cultures were picked with a sterile wire loop and suspended in 0.5 ml sterile, distilled water. The suspension was boiled for 30 minutes in a water bath. After cooling to room temperature, the preparation was centrifuged at 2000 x g and the supernatant harvested and stored at -20°C until analysis by polymerase chain reaction (PCR). PCR was first undertaken to confirm *Campylobacter* genus for the isolates after which three specific species were also identified: *C. coli*, *C. jejuni* and *C. lari*. The Campylobacter DNA preparation (2 µl) was amplified in a 25 µl reaction mix by mixing 2.5 µl 10X PCR buffer (Coraload), 0.5 µl dNTPs, 0.125 µl Taq DNA polymerase (Inqaba biotec, Pretoria, South Africa) and 0.1 µl of each specific primer to 10 pmole (Inqaba Biotec, Pretoria, South Africa), 2 µl DNA template and 18.657 µl DNAse/RNAse-free distilled water. The DNA was amplified using a program of initial heating at 94°C for 5 min followed by 30 cycles of denaturation at 94°C for 1 minutes, annealing at 56°C for 1 min, extension at 72°C for 1 min with a final extension of 72°C for 10 min using a Veriti 96 wells thermocycler, (Applied Biosystems, model 9902, Singapore) in 0.2-ml PCR tubes. The PCR products were kept at -20°C until gel electrophoresis was done.

The Campylobacter genus-specific primers, C412F and C1228 R, described by Linton *et al*. ^29^ were used to amplify a 812 bp fragment within the 16S rRNA gene of Campylobacter species using forward primer C412F 5’-GGATGACACTTTTCGGAGC-3’ and reverse primer; C1228R 5’-R-CATTGTAGCACGTGTGTC-3’. Multiplex PCR was carried out for *C. jejuni* and *C. coli* with specific primers CjejlpxAF, CjejipxAR (shared by both species) and CcollpxAF, described by Klena *et al*. ^15^ to amplify 331 bp and 391 bp fragment flanking the *lpxA* gene. The primer sequences were; CjejlpxAF (forward) 5’-ACAACTTGGTGACGATGTTGTA-3’, CjejipxAR (reverse, shared by CjejlpxA and CcollpxA) 5’-CAATCATGDGCDATATGASAATAHGCCAT-3’ for *C. jejuni* and for *C. coli* CcollpxAF (forward) 5’-AGACAAATAAGAGAGAATCAG -3’. The *C. lari* specific primers were forward primer lpxAC *,* 5’-AGACAATAAGAGAGAATCAG-3’ and reverse primer lpxARKK2M, 5’CAATCATGDGCDATATGASAATAHGCCAT-3’.

The PCR products were visualized by electrophoresis in a 1.5% agarose (Genetics analysis grade, Fisher Scientific, New Jersey) gel stained with 0.02% ethidium bromide and amplicons identified against molecular marker (50 bp DNA ladder, England Biolab) run alongside the samples.

For confirmation, the positively identified PCR products were submitted for sequencing. The PCR products were fist purified using exonuclease1, shrimp alkaline phosphatase mixture (ExoSAP mix) according to the manufacturer’s instructions. Briefly, this was done by adding 2.5 µl of ExoSAP mix to 10 µl PCR product. The mixture was then incubated at 37°C for 30 minutes and reaction stopped by heating at 95°C for 5 minutes. The clean PCR product was then quantified using a fluorimeter (Qubit 2.0, Invitrogen, USA). The clean DNA was first labelled with BigDye terminator v3.1kit (Applied Biosystem, CA, USA) according to the manufacturer’s instructions and loaded into Genetic Analyzer (ABI 3730 capillary analyser; Applied Biosystems, Foster City, CA, USA) for sequencing. Sequences were obtained in ABI files that were opened and edited to remove unspecific ends using BioEdit version 7.0.4 (Hall, CA, USA) software. Clean sequences were then submitted to NCBI GenBank database and BLASTn program used to test for homology and genetic identity of bacteria isolates.

***Antimicrobial sensitivity test (AST) for PCR-confirmed Campylobacter spp..*** *Campylobacter* spp. isolates were phenotypically tested for resistance using selected antimicrobial agents according to European committee on antimicrobial susceptibility testing (EUCAST) ^30^. Only antibiotics with EUCAST established breakpoints were tested, namely tetracyclines (tetracycline 30 mg), quinolones (ciprofloxacin 5 mg, naladixic acid 30 mg) and macrolides (erythromycin 15mg). Mueller-Hinton agar plates plus 5% de-fibrinated horse blood with 20 mg/L β-nicotinamide adenine dinucleotide Mueller-Hinton fastidious (β-NAD (MH-F)); (Oxoid, Basingstoke, UK) were prepared and dried at 35°C, with the lid removed, for 15 min prior to inoculation to reduce swarming. Inoculum turbidity was adjusted to McFarland 0.5 prior to inoculation. The antibiotic discs were placed on the inoculated plates using a sterile multi-disc dispenser and incubated in a microaerobic environment at 41±1°C for 24 hours. Isolates with insufficient growth after 24 hours of incubation were re-incubated immediately and inhibition zones read after a total of 40–48 hours incubation. The inhibition zones were defined by the point showing no growth when viewed from the front of the plate with the lid removed and with reflected light.

***Genotypic characterization of Campylobacter spp. isolates for antimicrobial resistance.*** A total of 90 antibiotic resistant, *Campylobacter* spp. isolates including; 11 *C. lari*, 30 *C. coli* and 49 *C. jejuni* were selected and characterized with PCR for demonstration of genes encoding resistance to tetracyclines including *tet*(A), *tet*(B), *tet*(C) and *tet*(O). Multiplex PCR was carried out as described above. Primers used for amplification of products encoding for the resistant genes to tetracyclines are shown in Table 1.

**Table 1.** Primers used for identifying tetracyclines encoding genes in selected bacteria isolates.

Primer sequence 5’-3’	Direction	PCR product, bp	genes	Reference
GTGAAACCCAACATACCCC	Forward	577	*Tet*(A)	31
GAAGGCAAGCAGGATGTAG	Reverse			
CCTCAGCTTCTCAACGCGT	Forward	635	*Tet*(B)	31
GCACCTTGCTGAGACTCTT	Reverse			
ACTTGGAGCCACTATCGAC	Forward	880	*Tet*(C)	32
CTACAATCCATGCCAACCC	Reverse			
AACTTAGGCATTCTGGCTCAC	Forward	515	*Tet*(O)	33
TCCCACTGTTCCATATCGTCA	Reverse			

## Results

**Figure 1.**
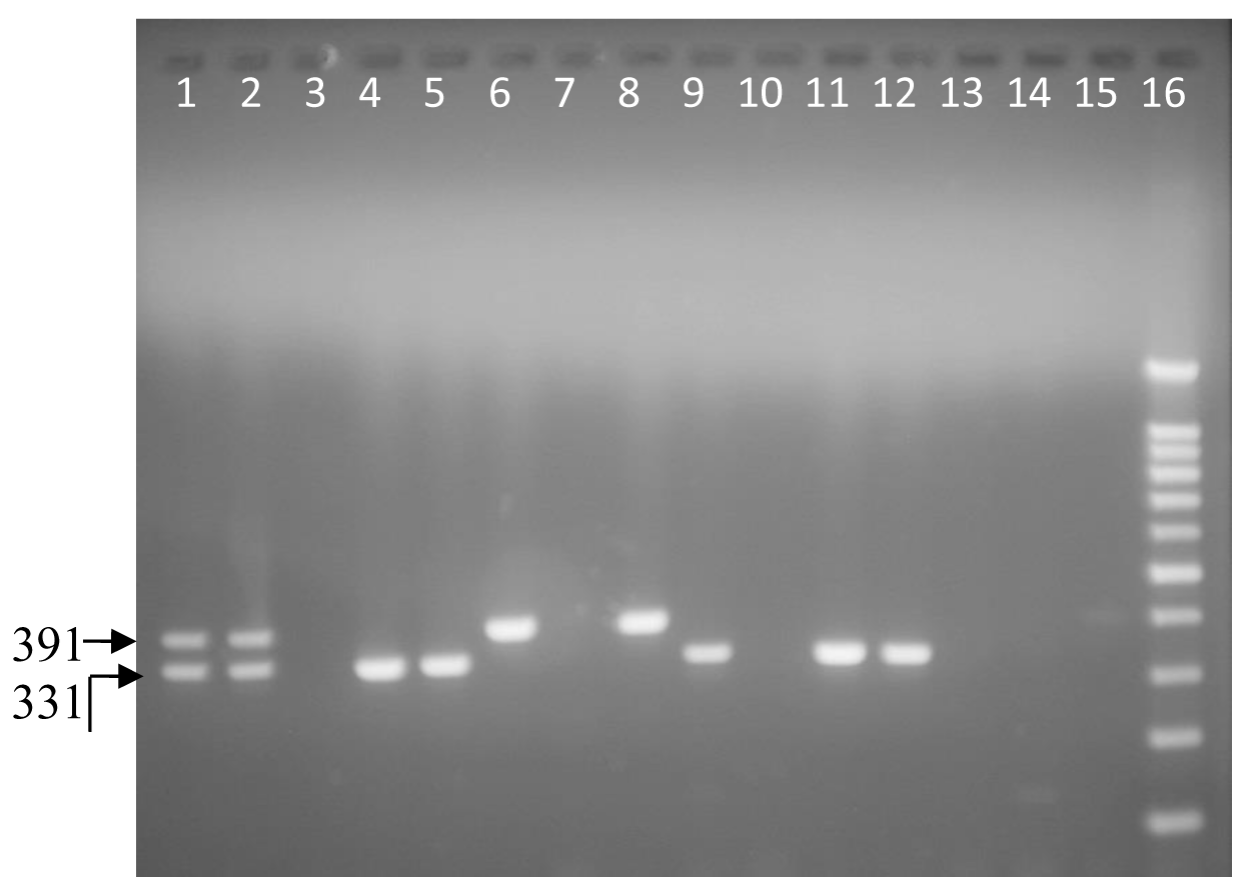
Ethidium bromide stained 1.5% agarose gel electrophoresis of *Campylobacter coli* (391 bp) and *C. jejuni* (331 bp) in a multiplex PCR with a 100-bp ladder. From left to right, lane 1 and 2 positive samples; mixture of *Campylobacter jejuni* and *Campylobacter coli* obtained from sequenced laboratory isolates (PHPT 1 &2). Lane 3 negative control: purified water. Lanes 4, 5, 9, 11 and 12: *C. jejuni*. Lanes 6, 8 and 15: *Campylobacter coli*. Lanes 7, 10, 13 and 14: negative samples. Lane 16: 100-bp ladder.

Of the 580 stool samples collected in 11 schools in Kibera, 294 (51%) were phenotypically characterized as suspect *Campylobacter* spp. When these isolates were subjected to PCR using genus and species-specific primers, 106 (18%) isolates were confirmed to be *Campylobacter* spp. Among the 106 isolates, 28 (4.8%) were *C. coli*, 44 (7.6%) *C. jejuni* ( Figure 1) while 11 (1.9%) were *C. lari*. In total, 23 (4.0%) *Campylobacter* isolates were not species identified as belonging to either *C. coli*, *C. jejuni* or *C. lari* ( Table 2).

**Table 2.** Molecular characterization by polymerase chain reaction of *Campylobacter* spp. isolates from school going children’s stool samples.

School	*C. coli*	*C. jejuni*	*C. lari*	Other *C.* spp.	Total *Campylobacter* spp.
A	0	0	0	5	8.5% (5/59)
B	3	0	2	3	21% (8/38)
C	0	0	2	3	8.1% (5/62)
D	6	7	0	2	34% (15/44)
E	8	9	1	2	25% (20/79)
F	0	3	0	1	21% (4/19)
G	5	10	1	2	23% (18/80)
H	0	3	1	1	9.4% (5/53)
I	0	4	1	0	17% (5/30)
J	5	6	2	2	22% (15/69)
K	1	2	1	2	13% (6/47)
Total	28	44	11	23	106
Prevalence	4.8% (28/580)	7.6% (44/580)	1.9% (11/580)	3.9% (23/580)	18.3% (106/580)

### Antimicrobial sensitivity test (AST) for confirmed *Campylobacter* spp.

The EUCAST disk diffusion method was used to determine the resistance patterns of only the identified isolates, 68 ( *C. jejuni, C. coli* and *C. lari*) confirmed by PCR ( Table 2). Fifteen isolates were not recovered from storage culture after identification and thus not tested *.* All of the antibiotics studied had isolates showing resistance towards them, with 96% of isolates resistant to tetracycline (30 mg), 93% to naladixic acid (30 mg) and all the isolates tested resistant to erythromycin (15 mg). The antibiotic that most isolates were sensitive to was ciprofloxacin (5 mg) which still had 84% of the isolates showing resistance ( Table 3). Of the four *tet* genes tested, *tet*(A) was most frequently identified in 20 (29.1%) of the isolates followed by *tet*(O) in 8 (11.7%) isolates and *tet*(C) in only 2 (2.9%) isolates. None of the isolates had more than one *tet* gene demonstrated. ( Table 3).

**Table 3.** Drug resistance patterns of pathogenic *Campylobacter* spp. isolates from school children’s stool samples, n=68 using EUCAST disk diffusion method (2016) and presence of genes encoding tetracycline resistance.

Antimicrobial agent	Resistance genes (no. of isolates)	Resistant isolates (EUCAST, 2016)			
		*C. jejuni* (n=30)	*C. coli* (n=27)	*C. lari* (n=11)	Total Resistance (%)
Tetracyline (30 mg)	*Tet*(A) (20), *tet*(B) (0), *tet*(C) (2), *tet*(O) (8)	30	26	11	67 (96)
Ciprofloxacin (5 mg)	Genotyping not done	25	23	9	57 (84)
Naladixic acid (30 mg)		29	24	10	63 (93)
Erythromycin (15 mg)	Genotyping not done	30	27	11	68 (100)

### Multidrug resistant profiles in *Campylobacter* spp. isolates

Four MDR profiles were observed. All of the tested isolates were resistant to two or more antimicrobial agents, but the majority of isolates (84%) were resistant to all the antibiotics studied (profile 3 and 4). *Campylobacter jejuni* had the highest number of isolates that were MDR with 25 (37%) isolates being resistant to all four antibiotics tested (profile 1). *C. coli* had 23 (34%) isolates resistant to all the four antibiotics while *C. lari* had 9 (13%) isolates resistant to the four antibiotics. One (1.5%) *C. jejuni* and *C. lari* isolates was resistant to drugs in profile 2, while three (3%) *C. coli* isolates were in this profile. However, profile 4 had only one (1%) *C. coli* isolate while profile 4 had 2 (3%) *C. lari,* 4 (6%) *C. coli* and 5 (7%) *C. jejuni* MDR isolates ( Table 4).

**Table 4.** Multidrug resistance (MDR) *Campylobacter* spp. isolates profile by antimicrobial sensitivity testing.

Drug (dose) profiles	No of MDR resistant isolates per species			MDR *Camylobacter* spp. isolates (n=68)
	*C. jejuni* (n=30)	*C. coli* (n=27)	*C. lari* (n=11)	
1. Ciprofloxacin (5 mg), nalidixic acid (30 mg), tetracycline (30 mg), erythromycin (15mg)	25	23	9	57 (84%)
2. Nalidixic acid (30 mg), tetracycline (30 mg), erythromycin (15 mg)	1	3	1	5 (7.3%)
3. Ciprofloxacin (5 mg), erythromycin (15mg)	5	4	2	11 (16%)
4. Tetracycline (30 mg), erythromycin (15 mg)	0	1	0	1 (1.5%)

## Discussions

A prevalence of 18% *Campylobacter* spp. in asymptomatic school going children was confirmed in this study. *Campylobacter* isolation from healthy children has been reported in developing countries ^34^ at a prevalence of 15%, which closely agrees with this study’s findings. The authors attributed the infections with *Campylobacter* to close contact with reservoir animals like chickens, as well as poor sanitation ^20^. Both of these factors are prominent in this study area, where chicken share housing with humans. The isolates were further characterized and *C. jejuni* was isolated more frequently (7.6%) as compared to *C. coli* (4.8%) and *C. lari* (2%), whereas 4% were none of the three species analysed. This distribution between *Campylobacter* species agrees with other reports from both developed and developing countries, including Kenya ^34– 36^. Among the thermophilic *Campylobacter* species, *C. upsaliensis* was not characterised using PCR in this study.

The *Campylobacter* spp. resistant to tetracycline had more *tet*(A) genes than *tet*(O) genes which were found in 20 (29%) and 8(12%) isolates respectively. This is consistent with Nguyen *et al*. ^20^ who identified more *tet*(A) genes than *tet*(O) genes in Kenyan *Campylobacter* spp. isolates from chickens, at 35% and 13% respectively. The high resistance rates obtained in this study, with 84% of isolates being resistant to all four agents, was in agreement with the findings of Nguyen *et al*. ^20^ and Coker *et al*. ^34^ for chicken and human *Campylobacter* isolates, respectively. Both studies reported more than 70% resistance to ciprofloxacin, nalidixic acid and tetracycline. However, these results contrast with those on human *Campylobacter* from diarrhoea cases in Western Kenya, where resistance to ciprofloxacin were observed in 6% cases, to nalidixic acid in 26%, and to tetracycline in 18%. Erythromycin resistance in this study was also high, in contrast to the findings of Nguyen *et al*. ^20^ in chicken-isolated *Campylobacter*. In the setting of the current study, with domestic animals hosted within the human settlements and poor sanitation, the possibility of cross-infection is very likely, as is horizontal transfer of antimicrobial resistance-encoding genes. Ciprofloxacin and erythromycin are the drugs of choice for *Campylobacter* treatment. These drugs are often used in Kenya for self-treatment of infections other than gastroenteritis, and resistance can be expected to increase in developing countries ^34^.

In conclusion, 18% asymptomatic school going children in the study area were found to be carriers of *Campylobacter coli*, *Campylobacter jejuni* and *Campylobacter lari*, 84% of these were multidrug resistant. More work on children carrying Campylobacter is needed to establish possibilities of previous exposure and virulence patterns of the Campylobacter isolates need to be investigated. Multidrug resistance in Campyobacter need to be addressed at all levels, the World Health Organization has recommended a multi-tiered and goal-oriented approach to control *Campylobacter* infections in both human and animals. Appropriate measures need to be taken to prevent Campylobacter transmission, including contaminated water and milk, through chlorination and pasteurization, respectively. Poultry, as the major reservoir, must be the main target in addressing human Campylobacteriosis ^4^.

## Data availability

Figshare: Multidrug resistant Campylobacter jejuni, Campylobacter coli and Campylobacter lari isolated from asymptomatic school going children in Kibera slum, Kenya.xlsx. https://doi.org/10.6084/m9.figshare.11302292 ^37^.

File ‘Multidrug resistant Campylobacter jejuni, Campylobacter coli and Campylobacter lari isolated from asymptomatic school going children in Kibera slum, Kenya.xlsx’ contains the bacterial species identified from samples, the antibiotic zones of inhibition and the presence or absence of antibiotic-resistance genes in each sample.

Data are available under the terms of the Creative Commons Attribution 4.0 International license (CC-BY 4.0).
