# Supplementary material for: Multidrug-resistant Campylobacter jejuni, Campylobacter coli and Campylobacter lari isolated from asymptomatic school-going children in Kibera slum, Kenya
Source: F1000Res. 2020 Sep 4;9:92. Originally published 2020 Feb 7. [Version 2] doi: 10.12688/f1000research.21299.2 (PMC10285328; doi:10.12688/f1000research.21299.2)
Supplement: Peer review history

# Peer review: Reviewer response for version 2

**DOI:** 10.5256/f1000research.29094.r176399

**Published:** 2023-06-13

**Authors:** Maria Pavlova

**Affiliations:** 1 National Center of Infectious and Parasitic Diseases, Sofia, Bulgaria

The conclusions are partly drawn and inadequately supported by the results. It is needed data about the contact people of Campylobacter-positive children. Are they examined for Campylobacter? Are only the children in their families positive for Campylobacter or any of their relatives?

 The second essential point is the reservoir of Campylobacter sp. How many of the Campylobacter carriers share a home space with chickens? And if Yes, why these chickens weren't examined to confirm a relatedness between the Campylobacter strains? Have Campylobacter-positive confirmed children a diarrhoea history soon before the research? 

 I advise the authors to compare the laboratory-confirmed campylobacter incidences with the laboratory-confirmed carriers by season, sex and age distribution, administrative areas etc. To present more epidemiological data and analysis.

 And second piece of advice are to present more suggestions for the reason for such a high tetracycline resistance- mass usage in feeding farm animals or mass usage in human medicine etc.

Is the work clearly and accurately presented and does it cite the current literature?

Yes

If applicable, is the statistical analysis and its interpretation appropriate?

Yes

Are all the source data underlying the results available to ensure full reproducibility?

Yes

Is the study design appropriate and is the work technically sound?

Yes

Are the conclusions drawn adequately supported by the results?

Partly

Are sufficient details of methods and analysis provided to allow replication by others?

Yes

Reviewer Expertise:

Enteric infectious diseases, diagnosis, outbreak surveillance & management, AMR.

I confirm that I have read this submission and believe that I have an appropriate level of expertise to confirm that it is of an acceptable scientific standard, however I have significant reservations, as outlined above.

# Peer review: Reviewer response for version 2

**DOI:** 10.5256/f1000research.29094.r70872

**Published:** 2020-10-12

**Authors:** Musafiri Karama

**Affiliations:** 1 Veterinary Public Health Section, Department of Paraclinical Sciences, Faculty of Veterinary Science, University of Pretoria, Onderstepoort, South Africa

The paper is scientifically sound in its current form and only minor, if any, improvements are suggested

Is the work clearly and accurately presented and does it cite the current literature?

No

If applicable, is the statistical analysis and its interpretation appropriate?

Yes

Are all the source data underlying the results available to ensure full reproducibility?

Yes

Is the study design appropriate and is the work technically sound?

Yes

Are the conclusions drawn adequately supported by the results?

No

Are sufficient details of methods and analysis provided to allow replication by others?

Yes

Reviewer Expertise:

Molecular epidemiology of Foodborne pathogens: Pathogenic E. coli, Campylobacter and Salmonella and bacteriophages.

I confirm that I have read this submission and believe that I have an appropriate level of expertise to confirm that it is of an acceptable scientific standard.

# Peer review: Reviewer response for version 1

**DOI:** 10.5256/f1000research.23456.r65988

**Published:** 2020-07-21

**Authors:** Byeonghwa Jeon

**Affiliations:** 1 Division of Environmental Health Sciences, School of Public Health, University of Minnesota, St. Paul, MN, USA

**Abstract:**

1. Background: Should read: the antibiotic resistance patterns of
2. Results: antibiotic concentrations, not the amount, need to be used. This applies to the entire manuscript. 

**Introduction:**

1. "It was not until recently that *Campylobacter* spp. was shown to exhibit multidrug resistance (MDR). Before that, the bacteria were considered to be susceptible" it may be difficult to say like this as the MDR was reported more than two decades ago. 
2. Quinolone resistance determination region (QRDR).
3. Campylobacter does not have parC. Mutations in parC cause FQ resistance in E. coli and Salmonella; but this is not applied to Campylobacter.

**Method:**

1. Antibiotic concentrations, not the amount, are to be made known in the supplement.  
2. Campylobacter is microaerophilic.  'Anaerobic conditions' --> 'Microaerobic conditions'.
3. Did the authors include a quality control strain to the antibiotic susceptibility test? 

**Results:**

1. Figure 1: There is no positive control for C. lari

**Others:**

1. Did the asymptomatic children carrying Campylobacter have any previous experience of diarrhea? It may be an important point to discuss whether Campylobacter-positive children are completely asymptomatic carriers or were recovering from Campylobacter exposure without manifesting clinical symptoms.
2. Overall, it reads well, but there are some minor English issues in some parts. 

Is the work clearly and accurately presented and does it cite the current literature?

Yes

If applicable, is the statistical analysis and its interpretation appropriate?

Not applicable

Are all the source data underlying the results available to ensure full reproducibility?

No source data required

Is the study design appropriate and is the work technically sound?

Partly

Are the conclusions drawn adequately supported by the results?

Partly

Are sufficient details of methods and analysis provided to allow replication by others?

Yes

Reviewer Expertise:

Antibiotic resistance of foodborne pathogens

I confirm that I have read this submission and believe that I have an appropriate level of expertise to confirm that it is of an acceptable scientific standard, however I have significant reservations, as outlined above.

# Peer review: Reviewer response for version 1

**DOI:** 10.5256/f1000research.23456.r59720

**Published:** 2020-03-27

**Authors:** Musafiri Karama

**Affiliations:** 1 Veterinary Public Health Section, Department of Paraclinical Sciences, Faculty of Veterinary Science, University of Pretoria, Onderstepoort, South Africa

The manuscript entitled “Multidrug-resistant *Campylobacter jejuni, Campylobacter coli and Campylobacter lari* isolated from asymptomatic school-going children in Kibera slum, Kenya” investigated the occurrence and antimicrobial resistance of *Campylobacter* spp. among school-going children in a populous slum of Kenya. The study is well executed but will need wordsmithing to improve its readability.

 A weakness of this manuscript is the lack of recent citations in the reference list. I suggest the authors update the reference list by including at least more than 60% of references that have been published in the last 10 years or less.  The conclusion will also have to focus on the topic of the research (Campylobacter prevalence and antimicrobial resistance in young children).

 I have inserted in the manuscript my corrections and suggestions to improve its clarity and style. This can be found here.

Is the work clearly and accurately presented and does it cite the current literature?

No

If applicable, is the statistical analysis and its interpretation appropriate?

Yes

Are all the source data underlying the results available to ensure full reproducibility?

Yes

Is the study design appropriate and is the work technically sound?

Yes

Are the conclusions drawn adequately supported by the results?

No

Are sufficient details of methods and analysis provided to allow replication by others?

Yes

Reviewer Expertise:

Molecular epidemiology of Foodborne pathogens: Pathogenic E. coli, Campylobacter and Salmonella and bacteriophages.

I confirm that I have read this submission and believe that I have an appropriate level of expertise to confirm that it is of an acceptable scientific standard, however I have significant reservations, as outlined above.
